# On the Respiration of the Alligator, and Mrs. Willard’s Theory of the Circulation

**Published:** 1852-08

**Authors:** Albert Welles Ely

**Affiliations:** New Orleans


*On the Respiration of the Alligator, and Mrs. Willard's Theory of the Circulation.* By Albert Welles Ely, A. M., M. D., of New Orleans.—In the case of the alligator, vivisected on the 20th of August, 1851, which made Dr. Cartwright a convert to the new theory, and caused him "to become one of the standard-bearers of the *Filia nata Jovis** of the New World," we doubt whether the alligator was really asphyxiated; for the ligature was not about his trachea more than an hour. Dr. Dowler says about 30 or 40 minutes; Prof. Forshey 20 minutes; and Dr. Cartwright about an hour. Now, how is it possible that the alligator in question could have been asphyxiated, or stone dead, or dead at all, after having a ligature about his trachea for only one hour, at most; when it is well known that alligators can remain under water almost any length of time? They are known to lie at the bottom of lakes and pools of water whole days, without coming out to take the air. How, then, can an alligator be killed by tying a ligature about his trachea? Undoubtedly they become stupid on the application of the ligature, and that they remain so for a time. When they go to the bottom of lakes, they lie there perfectly still and stupid, appa-

---

*Mrs. Willard has assuredly too much good sense not to disclaim thi inflated compliment of Dr. C.

rently, until, after a long time, they arouse themselves and come out.

But we have proof positive on this point. We know, positively, that the ligature about an alligator's trachea for one hour only, will not kill him. I will cite a case: On the 6th of May last, the trachea of an alligator was tied by Dr. Dowler, in the presence of others of this this city, for the express purpose of repeating Dr. Cartwright's experiment of inflating the lungs, made on the 20th of May, 1851. The animal became stupid, like the other, at first; but just as they were preparing to inflate, the animal, who appeared dead, suddenly revived *of his own accord*, and fought the by-standers most desperately, *in spite of the wicked ligature about his trachea!!*

What, then, becomes of Dr. Cartwright's statement, that "it is a remarkable fact that tying the trachea is the only means by which that animal can be expeditiously killed?" And what, too, becomes of the proof, afforded by the experiment of the 20th of August, 1851, of Mrs. Willard's theory? Is it not highly probable, that that alligator was no more suffocated to death than its brother martyr to the cause of science of the 6th of May, 1852? Undoubtedly it would have revived, with the ligature about its trachea, if let alone. If Dr. Cartwright had suffered the alligator to remain undisturbed a little longer, he would have been spared the pleasure of recording the apotheosis of Mrs. Willard, and of congratulating her on the probable happy result of her new theory—the discovery of "the art, so long sought by the ancients, of making the old younger, children healthy, men vigorous, and women pretty."*

Dr. Cartwright will please excuse me for my seeming incredulity, for I am never over-disposed to take too much for granted. His Boston opponents, who probably never saw an alligator in their lives, are excusable for not questioning the data of the case.

Dr. Cartwright states, that the heart of the alligator (vivisected on the 20th of August, 1851, by Dr. Dowler,

---

*See Dr. Cartwright's letter in the Boston Med. Journal of January 14, 1852.

and upon which alone he bases the "demonstration" of Mrs. Willard's theory) was dead and cold—that he took it in his hand—that not a motion or sign of life occurred. We do not question that he took the heart in his hand, and that it felt cold. He probably knows that the heart of an alligator always feels cold; but as to the heart having ceased to contract, in an alligator like that, just killed, we think there must be some mistake. The heart of the alligator is the last thing to die. It will contract itself for hours after being entirely removed from the body. Neither Dr. Dowler nor Dr. Nutt, who were present, observed that the heart was dead, although Dr. Dowler performed the dissection. These facts, together with another, that Dr. Cartwright did not write his account of the experiments to Mrs. Willard until some months had elapsed, leave room for supposing that there is some mistake about the heart being dead. We think it could not be so. The heart of an alligator, five or six feet long, is small, and contracts slowly and at considerable intervals. If the heart still contracted, in spite of the ligature about the trachea, the circulation must have been going on, and therefore the inflation of the lungs did not start the circulation.

The circulation of the alligator is independent of respiration, as is proved by its being able to remain under water for whole days, and by the experiment of the 6th of May last, in which the alligator, with his trachea tied, revived of himself, after lying stupid about half an hour. The structure, too, of the circulatory system, in the alligator, is precisely such as would lead one to suppose that he could live without breathing, and even with a ligature about his trachea. About one half of the blood in the alligator circulates constantly through his system, without ever going to the lungs at all. His structure is such, that it appears to be optional with him whether his blood be ærated or not. Like all other animals, he can live as long as his blood circulates, and this must take place, whether he is at the bottom of lakes or basking in the sun on their shores.—*N. O. Med. & Surg. Jour.*

